# Is there a dose-dependent effect of genetic susceptibility loci for gastric cancer on prognosis of the patients?

**DOI:** 10.18632/oncotarget.13123

**Published:** 2016-11-04

**Authors:** Lei Cheng, Li-Xin Qiu, Ming Jia, Fei Zhou, Meng-Yun Wang, Ruo-Xin Zhang, Yajun Yang, Xiaofeng Wang, Jiucun Wang, Li Jin, Qing-Yi Wei

**Affiliations:** ^1^ Cancer Institute, Collaborative Innovation Center for Cancer Medicine, Fudan University Shanghai Cancer Center, Xuhui, Shanghai, China; ^2^ Department of Oncology, Shanghai Medical College, Fudan University, Xuhui, Shanghai, China; ^3^ Department of Medical Oncology, Fudan University Shanghai Cancer Center, Xuhui, Shanghai, China; ^4^ Ministry of Education Key Laboratory of Contemporary Anthropology and State Key Laboratory of Genetic Engineering, School of Life Sciences, Fudan University, Shanghai, China; ^5^ Fudan-Taizhou Institute of Health Sciences, Taizhou, Jiangsu, China; ^6^ Duke Cancer Institute, Duke University Medical Center, and Department of Medicine, Duke University School of Medicine, Durham, North Carolina, USA

**Keywords:** gastric cancer, genome-wide association study, genetic risk score, prognosis

## Abstract

Literature suggests that genetic variants associated with increased susceptibility to gastric cancer (GCa) are mostly located in genes involved in carcinogenesis and possibly tumor progression. Therefore, we hypothesize that high genetic susceptibility is also associated with prognosis of the patients. To test this hypothesis, we selected a total of 42 common genetic variants that were reportedly associated with GCa risk with a high level of evidence obtained from either genome-wide association studies (GWASs) or meta-analyses and performed survival analysis of patients used in a case-control analysis. We first used 1115 GCa cases and 1172 cancer-free controls of ethnic Han Chinese to construct a weighted genetic risk score (GRS). Then, we included 633 GCa cases with available clinical information, fit GRS in a fractional polynomial Cox proportional hazards regression model to investigate whether there is a dose-dependent effect of GRS on risk of death in survival analysis. Dynamic predictive value of genetic risk for prognosis was also calculated. The results showed that the increase of GRS had no effect on risk of death in these GCa patients. Compared with GCa patients with the medium GRS, there was no significant difference in survival in patients with either a low (*P =* 0.349) or a high (*P =* 0.847) GRS. The results unchanged when data were stratified by tumor stage and Laurens classification. Time-dependent predictive value for prognosis in considering both clinical factors and GRS was comparable with that in considering clinical factors alone, for either all patients (*P =* 0.986) or stage- and Laruen type-based subgroups (*P >* 0.05 for all). In conclusion, higher polygenic susceptibility loci for GCa may not indicate worse prognosis of Chinese patients. Additional variants of relevant genes modulating GCa patients survival need to be further identified.

## INTRODUCTION

Gastric cancer (GCa) is the second commonly diagnosed cancer and also the second leading cause of cancer-related deaths in China [1]. According to recent cancer statistics, there were approximately 679,100 new cancer cases and 498,000 new deaths in 2015 in China, accounting for 15.8% of all cancer cases and 17.7% of all cancer deaths, respectively. Before diagnosis, GCa patients in the early stage did not have obvious symptoms usually, but the disease rapidly progressed, if its diagnosis was missed. Therefore, only prevention and earlier detection/diagnosis of GCa will reduce the incidence and mortality of GCa in China. Currently, the major treatment for GCa is still surgery, if the tumors are resectable at the time of diagnosis [2]. However, the high recurrence rate makes it the necessary to identify additional markers for prognosis, which may facilitate individualized treatment and management. Some clinical factors, such as Lauren's classification, have been shown to be associated with GCa prognosis. For example, patients with intestinal type tumors may have a better prognosis than those with diffuse type tumors [3, 4]; however, there is lack of promising genetic predictors for GCa prognosis [5].

As a result of rapid advance in genomic technologies, genetic variants have been demonstrated by previous studies as potential factors that may affect both cancer etiology [6] and patients’ response to therapies [7] . For example, single nucleotide polymorphisms (SNPs), presented more than 1% in the general population, have been reported to be associated with GCa risk [8–10]. These findings provide tools for early detection, diagnosis and prevention at the genetic level in the at-risk populations. To date, five large published genome-wide association studies (GWASs) have revealed a series of genetic variants associated with susceptibility to GCa [11–15]. Some of these results have then been successfully reproduced by subsequent large case-control studies with sufficient statistical power [16, 17].

Recently, summary data on genetic susceptibility to GCa were pooled and comprehensively reviewed by a large meta-analysis [18]. Interestingly, it was reported that these GWAS-derived SNPs associated with GCa susceptibility are located in genes that are involved in carcinogenesis and tumor progression, which may affect the invasiveness and metastatic potential of GCa [19–21]. Moreover, the biological role of susceptibility genes in tumor progression may be reflected in the correlation of some susceptibility loci with poor prognosis [10, 22–25]. However, it has been reported that the somatic status of tumor cells is quit heterogeneous among patients, indicating that germline variants may also play a role in determining which somatic alterations is likely to be acquired [26], in addition to exposure to carcinogens. Therefore, tumors of patients with different inherited genetic background may act in different biological ways.

Taken together, there is one possibility that tumors in patients with different genetic risk of developing cancer derived from germline variants may have a different pattern of disease course. Therefore, we hypothesize that a well-defined genetic predisposition of patients with GCa is associated with patients’ prognosis. To test this hypothesis, we used the available genotyping data from an ongoing case-control study of GCa and survival data from the same patients in a single institution.

## RESULTS

**Figure 1:**
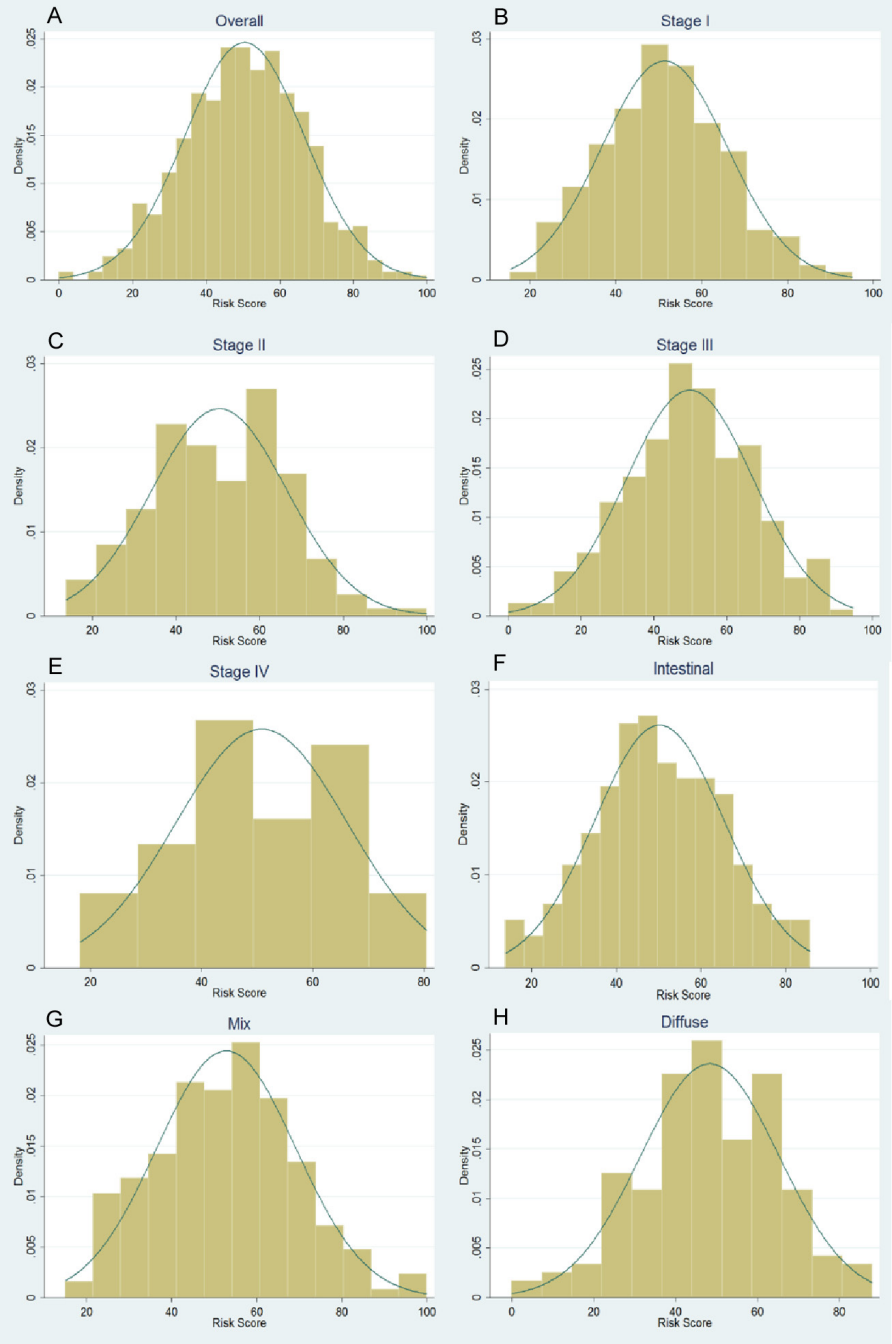
Normal distribution of genetic risk score (GRS) for overall patients (A), subgroup of stages (B-E) and Lauren's classification (F-H)

**Figure 2:**
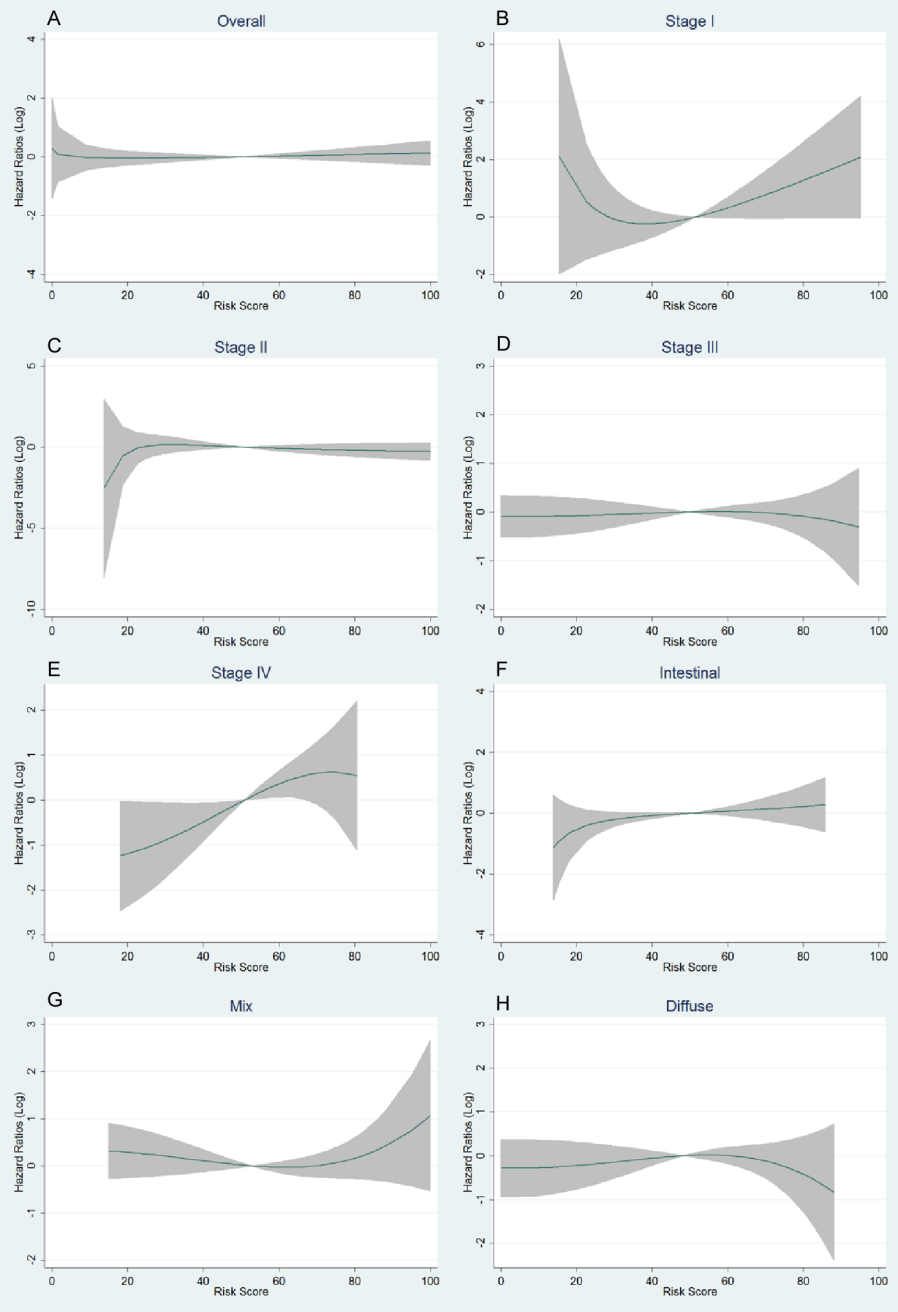
Trends of logarithmic HRs for death with the increase of genetic risk score (GRS) in multivariate fractional polynomial Cox proportional hazards regression models Overall patients **A**.; subgroup of stages **B**.-**E**.; subgroup of Lauren's classification **F**.-**H**. The continuous line is the point estimation of logarithmic HRs and the shadow area is the 95% confidence interval of logarithmic HRs.

**Figure 3:**
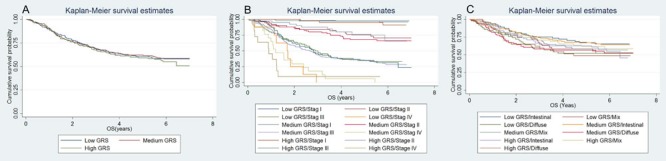
Kaplan-Meier (KM) curve for the survival comparison of patients with different GRS Overall patients **A**.; subgroup of stages **B**.; subgroup of Lauren's classification **C**.

Characteristics of the subjects are presented in Supplementary Tables 1 and 2. The 42 selected SNPs and their relevant information are presented in Supplementary Table 3. In the initial tests, the weighted genetic risk score (GRS) showed a normal distribution in all the subjects and subgroups as well (Figure 1). In the subsequent analyses, the results of multivariate fractional polynomial Cox proportional hazards regression models showed that the increase of GRS did not have an effect on the risk of death in the patients, although an upward trend was observed in the subgroup of stage IV and patients with Lauren's mix-type tumors (Figure 2). According to the segmented trend of fractional polynomial regression model, a low, medium and high GRS was defined as the < 25%, 25%-75% and >75% quartiles, respectively. However, compared with patients with the medium GRS, those with a low and high GRS did have a similar survival [low *vs* medium *vs* high corresponding 74% *vs* 61.71% *vs* 59.09% of 5-year overall survival (OS); log-rank test, *P* = 0.879, Figure 3], and the results remained unchanged after adjustment for age, sex, stage, Lauren's classification and differentiate degree of tumors, and treatment (low *vs* medium, HR = 0.87 and *P* = 0.349, and high *vs* medium, HR = 0.97 and *P* = 0.847). These results did not change substantially in the subgroups of either stage or Laruen's classification (Table 1). Moreover, GRS was not helpful, in addition to clinical factors, to discriminate more patients with different outcomes in either the overall patients [Concordance index (C-index): 0.78 *vs* 0.78, *P* = 0.986] or subgroups of stage and Lauren's classification (Table 2).

**Table 1:** Association of GRS with survival of GCa patients in an Eastern Chinese population

Group	GRS	Crude HR (95% CI), *P* value	Adjusted HR (95% CI)^a^, *P* value	Log-rank test^b^ for 5-year OS, *P* value
Overall	Low	1.01 (0.76, 1.36), 0.924	0.87 (0.64, 1.17), 0.349	60.74% vs 61.71% vs 59.09%, 0.879
	Medium	Reference	Reference	
	High	1.08 (0.81, 1.45), 0.603	0.97 (0.72, 1.31), 0.847	
Stage I	Low	0.66 (0.07, 5.94), 0.714	0.62 (0.06, 5.91), 0.677	97.44% vs 97.14% vs 95.00%, 0.546
	Medium	Reference	Reference	
	High	1.89 (0.42, 8.46), 0.404	1.97 (0.40, 9.63), 0.402	
Stage II	Low	0.86 (0.45, 1.64), 0.653	0.76 (0.49, 1.48), 0.425	76.00% vs 68.00% vs 75.00%, 0.804
	Medium	Reference	Reference	
	High	0.79 (0.39, 1.60), 0.506	0.73 (0.35, 1.53), 0.401	
Stage III	Low	0.91 (0.63, 1.32), 0.629	0.85 (0.59, 1.24), 0.413	34.85% vs 34.45% vs 34.92%, 0.880
	Medium	Reference	Reference	
	High	0.94 (0.65, 1.36), 0.750	0.90 (0.60, 1.27), 0.467	
Stage IV	Low	1.09 (0.45, 2.63), 0.848	0.91 (0.34, 2.41), 0.847	0% vs 5.88% vs 9.09%, 0.584
	Medium	Reference	Reference	
	High	1.39 (0.62, 3.09), 0.420	1.99 (0.74, 5.35), 0.171	
Intestinal	Low	0.96 (0.58, 1.59), 0.878	0.66 (0.39, 1.12), 0.128	67.19% vs 67.86% vs 62.71%, 0.425
	Medium	Reference	Reference	
	High	1.33 (0.83, 2.14), 0.239	1.15 (0.70, 1.88), 0.584	
Mix	Low	1.13 (0.66, 1.93), 0.659	1.06 (0.61, 1.86), 0.831	58.14% vs 58.33% vs 61.82%, 0.799
	Medium	Reference	Reference	
	High	0.92 (0.55, 1.54), 0.743	0.93 (0.54, 1.60), 0.802	
Diffuse	Low	1.06 (0.63, 1.77), 0.820	0.94 (0.54, 1.61), 0.812	52.00% vs 56.58% vs 48.65%, 0.930
	Medium	Reference	Reference	
	High	1.10 (0.63, 1.92), 0.749	0.97 (0.53, 1.78), 0.919	

GRS, genetic risk score; GCa, gastric cancer; HR, hazards ratio; OS, overall survival;

^a^Adjusted for gender, age, disease stage, Lauren classification, tumor differentiation and treatment;

^b^Log-rank test of 5-year OS, based on the comparison of Low versus medium versus high GRS.

**Table 2:** Dynamic predictive value (C-index) of considering clinical factors and GRS versus that of considering clinical factors alone in an Eastern Chinese population

Group	C-index	*P* value^b^
Overall	0.78 vs 0.78	0.986
Stage I	0.79 vs 0.77	0.880
Stage II	0.60 vs 0.60	0.999
Stage III	0.56 vs 0.56	0.999
Stage IV	0.68 vs 0.62	0.417
Intestinal	0.82 vs 0.81	0.812
Mix	0.75 vs 0.75	0.999
Diffuse	0.78 vs 0.78	0.991

GRS, genetic risk score; C-index, concordance index;

^a^Clinical factors include age, gender, disease stage, Lauren classification, tumor differentiation and treatment;

^b^*P* value for bootstrapping test (number of boots = 1000)

## DISCUSSION

Recently, it has been shown that there is no association between GRS of prostate cancer and risk of radiotherapy toxicity, although some susceptibility genes were indeed involved in cellular radiation response [27]. In the present study, we tested the hypothesis that GRS, a genetic estimation of GCa risk, is also associated with survival in an Eastern Chinese population. We first used a case-control study to develop a population-specific GRS based on 42 well-established GCa susceptibility loci derived from published GWASs and meta-analysis and then used this GRS in a survival analysis of the patients from the same case-control study. Consistently with the reported prostate cancer study, we did not find any association between genetic risk of GCa and prognosis of the patients in this study population, nor did we find any evidence for an interaction between GRS and stage or Laruen's classification, two clinical factors closely associated with prognosis of GCa. These data suggest that individuals who are susceptible to GCa are not necessarily have a poor clinical outcomes, which indicates that additional genetic variants that are responsible for poor clinical outcomes of GCa patients need to be further identified.

A number of studies indicated that a constitutional or germline background in cells, which is a primary genetic background inherited from parents, may indicate which somatic pattern in the tumor is more likely to be subsequently acquired. This phenomenon was further demonstrated in several cancer types. For example, it was reported that melanoma patients with germline variants in *MC1R* tended to acquire *BRCA1* mutations more likely than those without genetic variants in *MC1R* [28]. Moreover, breast cancer patients with inherited *BRCA1* variants have a higher frequency of copy number alterations than those without inherited *BRCA1* variants [29]. On the other hand, studies have found that SNPs associated with GCa risk may regulate the expression of their nearby genes. These genes, such as *PRKAA1* [14], *PLCE1* [12] and *MUC1* [30], also play an important role in cancer progression and tumorigenesis. For example, the *PRKAA1* gene encodes catalytic α-subunit of 5′ AMP-activated protein kinase, which is involved in protecting tumors from energy deprivation [31] and thus promoting cancer metastasis [32, 33]. As a susceptible gene for GCa and esophageal squamous cell carcinoma, the effect of *PLCE1* in tumorigenesis was also elucidated by a recent study, in which the *PLCE1* gene targeted by miR-145 impaired tumor metastasis and proliferation [19]. Besides, several studies reported a biological role of *MUC1* in cancer metastasis through disturbing cell adhesions [34–36]. Interestingly, the use of anti-MUC1 antibody was helpful for identifying poor differentiated cells in GCa tumors [20]. Tumorigenic effect of other susceptible genes, such as *PKLR*, on GCa was also reported [21]. Although all of these previous findings suggest a possibility that GCa patients with a different genetic susceptibility may have a different disease course that may contribute to the prognosis variation, we did not find any polygenic association with the prognosis of Chinese patients with GCa. The negative results can be explained as follows: first, not every germline variant plays an important role in determining which of somatic alterations is likely to be acquired, although some genetic variants acting as a key driver for somatic alterations have been identified by previous studies [28, 29]; second, environment factors may play a more important role in predicting prognosis than germline variants, which may have masked the effect of germline variants on prognosis of these GCa patients.

Previous studies have revealed a number of genetic variants associated with GCa prognosis. For example, SNPs of the *TYMS* gene, such as rs16430 and rs1059394, were found to be associated with GCa prognosis in North-American patients [37]. Genetic variants in genes involved in the nucleotide excision repair (NER) pathway were also shown to have an impact on survival of GCa patients in both Japanese and North-American patients [38]. Interestingly, a recent study found that the *PSCA* rs2294008 T allele was associated with either a poor prognosis or an increased susceptibility of Spanish GCa patients [10].

In the present study, GRS in the subgroup of stage IV patient showed the notable ability to help clinical factors to distinguish more patients with better and worse survival in a dynamic process (Cindex, 0.68 *vs* 0.62), although the result did not reach statistical significance (*P* = 0.417). However, the result from a limited sample size in stage IV patients (*n* = 36 only) without a sufficient statistical power needs to be validated in additional larger studies. Another interesting finding in the present study was that Lauren's classification had no effect on the association between genetic risk and disease prognosis. According to Lauren's classification, tumors can be defined as an intestinal, mix or diffuse type [39], and tumor cells with these three different Lauren's types may act in different biological ways. Therefore, it is valuable to detect any association of genetic risk of GCa with prognosis in different Lauren's types. Disease stage is another strong predictor for prognosis. However, it has the characteristic of time dependency, which is changeable in the development of cancer over time. Moreover, patients with an advanced stage may miss the opportunity of treatment due to the poor performance status. Because genetic risk is inherited and thus not changeable in the lifetime, elucidating the association between genetic risk and prognosis for GCa is helpful in making treatment decision at the time of presentation of the disease in different stages.

There are some limitations in the present study. First, due to a decrease in the number of patients in subgroup analysis, statistical power was greatly reduced. Second, although 42 well-established susceptibility loci selected from GWASs and confirmed in large meta-analyses were used, SNPs in some driver genes for tumorigenesis or information about somatic mutations in the same genes were missed in the analyses.

In conclusion, our results do not support our original hypothesis. It is likely that genetic risk of GCa, based at least on those susceptibility loci identified in previous GWASs and high-evidence meta-analysis, may not be associated with the prognosis, as we observed in the patients of an Eastern Chinese population. Due to limitations of the present study using retrospective data, these results needed to be validated by larger perspective studies. Likewise, our findings implicate that genetic risk of GCa may not affect the treatment decision in Chinese GCa patients, and additional genetic variants that may be associated with clinical outcomes in GCa patients need to be further identified.

## MATERIALS AND METHODS

### SNP selection

In the present study, the established susceptibility loci for GCa were selected by the following criteria: 1) GWAS-derived potential susceptibility loci that were reportedly associated with GCa risk [11–15]; and 2) susceptibility loci for GCa that was confirmed by a recent large meta-analysis as to be high-level evidence [18]. As a result, a total of 42 known SNPs were chosen for genotyping in the study population.

### Study subjects

To construct the GRS in a case-control study, we first used 1,115 unrelated ethnic Han Chinese patients with newly diagnosed and histopathologically confirmed primary GCa, who were recruited from Fudan University Shanghai Cancer Center (FUSCC) in Eastern China between January 2009 and March 2011. Patients with neuroendocrine tumors and gastric stromal tumors were excluded. Then, we included an additional 1,172 cancer-free ethnic Han Chinese controls matched the cases by age, sex, smoking and drinking-matched, who were recruited from the Taizhou Longitudinal (TZL) study conducted at the same time period in Eastern China. For the subsequent survival analysis, we used the 633 GCa patients with available clinical data. Disease stage was categorized by the 7th AJCC staging system. A written informed consent for donating biological samples to scientific research was provided by all the subjects whose blood samples were stored at the biobanks at both FUSCC and TZJ studies. The study protocol was approved by the FUSCC institutional review board.

### Genotyping and quality control

Blood samples of the GCa cases and controls were provided by the tissue banks at both FUSCC and TZL study. Demographic data of these subjects were collected. Clinical information with survival data was collected during the subsequent follow-up. DNA was extracted from blood samples, and the selected 42 SNPs were genotyped by Sequenom Mass Array in Mass ARRAY Analyzer 4 platform (Sequenom, CA, USA). One negative control and one duplicate sample were used for quality controls in each of the 96 plates. All the primers were designed by Assay Design Suite v2.0 from Mysequenom online software (https://www.mysequenom.com). Genotyping results of 5% patients were repeated, and the consistency was 100%.

### Statistical methods

Initially, we used the penalty function in the iterative sure independence screening (ISIS) algorithm, in which a best-fit logistic regression model was generated through regularized log likelihood for the variables picked by ISIS [40]. Then, a weighted GRS was calculated by coefficients in the best-fit logistic regression model as follows:

Where, i is the i th subject, j is the j th SNP, coefficientj is the coefficient for the j th SNP in the best-fit logistic regression model, and *n*ji is the risk allele dosage for the j th SNP in i th subject [27]. Then the GRS was translated to a range of 0-100, according to its distribution among the study subjects.

Then, fractional polynomial Cox proportional hazards regression model was used to evaluate the trends of logarithmic HRs for death with the increase of GRS. Overall survival was defined as the period between diagnosis and death. Survival data for patients alive or loss to follow-up were considered censored. Survival comparison was made by using the log-rank test and Kaplan-Meier curve. A dynamic predictive model for survival data, with the concordance index (C-index) as the estimation of the information in the predictive model, was adapted to reveal whether GRS for GCa was helpful, in addition to clinical factors, to discriminate more patients with different outcomes. The bootstrapping test was used for comparisons of the C-index.

All statistic work was achieved by R (version 3.2.4, R Foundation, Vienna, Austria) and STATA software (version 12, StataCrop, Texas, USA).

## SUPPLEMENTARY MATERIALS TABLES


