# Supplementary material for: Is there a dose-dependent effect of genetic susceptibility loci for gastric cancer on prognosis of the patients?
Source: Oncotarget. 2016 Nov 4;8(11):18435–43. doi: 10.18632/oncotarget.13123 (PMC5392341; doi:10.18632/oncotarget.13123)
Supplement: Supplementary file 1 [file oncotarget-08-18435-s001.pdf]

Is there a dose-dependent effect of genetic susceptibility loci for gastric cancer on prognosis of the patients?

Supplementary Material

Supplementary Table S1: Demographics of the subjects from a case-control study of gastric cancer included used in the first stage of the analysis

Variable	Case<br>No. (100%)	Control<br>No. (100%)	*P* value [a]
All subjects	1,115 (100.0)	1,172 (100.0)	
Age (year)			0.87
≤59	569 (51.0)	593 (50.6)	
>59	546 (49.0)	579 (49.4)	
Sex			0.61
Male	793 (71.1)	822 (70.1)	
Female	322 (28.9)	350 (29.9)	
Smoking			0.49
Yes	677 (61.2)	734 (62.6)	
No	430 (38.8)	438 (37.4)	
Drinking			0.66
Yes	261 (23.6)	267 (22.8)	
No	846 (76.4)	905 (77.2)	

[a] *P* value for Chi-square test

Supplementary Table S2: Clinical information of gastric cancer patients included in the second stage of the analysis

Variable	Case<br>No. (100%)
All patients	633
Age (year)	59.66± 11.35
≤ 59	297 (46.2)
>59	336 (54.8)
Sex	
Male	445 (70.3)
Female	188 (29.7)
Therapy	
Yes	452 (71.4)
No	181 (28.6)
Surgery	
Yes	597 (94.3)
No	36 (5.7)
Stage	
I-II	349 (55.1)
III-IV	284 (45.9)
Lauren classification	
Intestinal	263 (41.5)
Mix	194 (30.6)
Diffuse	163 (25.8)
Unsure	13 (2.1)
Tumor differentiation	
Well-moderate	147 (23.2)
Poor	486 (76.7)

Supplementary Table S3: SNPs selected for the analysis in this study

SNP	Chromosome	Gene	Position	Alleles transform	MAF[a]		HWE[b]
					Cases	Controls	
rs3834129	2	*CASP8*	201232809	CTTACT>DEL	0.214	0.194	0.140
rs78747266	2	*MTA3*	42725594	A>G	0.025	0.021	0.031
rs1108143	2	--	234557214	A>G	0.098	0.098	0.339
rs13361707	5	*PRKAA1*	40791782	C>T	0.442	0.531	0.755
rs2294008	8	*PSCA*	142680513	C>T	0.306	0.270	0.622
rs3087465	3	*TGFBR2*	30605668	G>A	0.197	0.184	0.524
rs4072037	1	*MUC1*	155192276	T>C	0.125	0.167	0.055
rs13042395	20	*SLC52A3*	773867	C>T	0.373	0.387	0.743
rs187150116	6	--	85691180	G>T	0.223	0.022	0.075
rs11754426	6	--	79399426	T>C	0.968	0.088	0.941
rs1695	11	*GSTP1*	67585218	A>G	0.188	0.195	0.396
rs3762272	1	*PKLR*	155291986	T>C	0.247	0.283	0.907
rs2274223	10	*PLCE1*	94306584	A>G	0.239	0.195	0.148
rs1799724	6	*TNF LTA*	31574705	C>T	0.125	0.128	0.650
rs75797460	1	--	197070213	G>A	0.191	0.101	0.954
rs9841504	3	*ZBTB20*	20938343	C>G	0.162	0.140	0.699
rs7712641	5	--	89607147	C>T	0.474	0.468	0.604
rs74402387	7	*ELMO1*	36962240	T>G	0.025	0.030	0.299
rs78826154	3	--	164388560	C>G	0.027	0.030	0.970
rs77065565	10	*GFRA1*	116257629	G>A	0.853	0.090	0.666
rs12499763	4	--	83910247	T>C	0	0	
rs139120256	3	*SEMA5B*	122948289	C>T	0.022	0.022	0.441
rs532454	11	*GDPD5*	75443022	T>C	0.028	0.223	0.415
rs851911	12	*FAM19A2*	61940578	A>C	0.060	0.059	0.286
rs2114910	12	*GAS2L3*	100608337	C>G	0.486	0.474	0.882
rs2294693	6	*UNC5CL*	41037763	T>C	0.342	0.251	0.688
rs2976392	8	*PSCA*	142681514	G>A	0.300	0.270	0.458
rs7839487	8	--	28608179	A>G	0.528	0.040	0.149
rs8176719	9	--	133257521	DEL>C	0.429	0.458	0.615
rs9908993	17	--	78637503	A>G	0.487	0.497	0.838
rs10074991	5	*PRKAA1*	40790449	G>A	0.442	0.536	0.956
rs12308015	12	--	94894879	T>C	0.287	0.295	0.005
rs13220767	6	*EYS*	64945266	C>T	0.002	0.004	0.882
rs17647701	15	--	92597110	G>A	0.692	0.066	0.004
rs61765798	1	--	18824073	A>G	0.113	0.114	0.247
rs72627187	14	*NRXN3*	78138183	T>C	0.313	0.329	0.319
rs74684850	16	*WWOX*	78235875	A>G	0.049	0.055	0.394
rs76845414	8	*TNFRSF10*	23042984	T>C	0.038	0.033	0.013
rs77283376	2	*LOC100506047*	43134421	G>T	0.091	0.087	0.931
rs79776715	9	--	30386556	T>C	0.036	0.031	0.0001
rs80142782	1	*ASH1L*	155515236	T>C	0.061	0.081	0.514
rs431387	19	--	16178165	C>T	0.0009	0.0009	0.977

[a]Minor allele frequency; [b]Hardy-Weinberg equilibrium test
